# Synthesis and Irreversible Thermochromic Sensor Applications of Manganese Violet

**DOI:** 10.3390/ma11091693

**Published:** 2018-09-12

**Authors:** Duy Khiem Nguyen, Quang-Vu Bach, Jong-Han Lee, In-Tae Kim

**Affiliations:** 1 Department of Civil Engineering, Pusan National University, 2, Busandaehak-ro 63beon-gil, Geumjeong-gu, Busan 46241, Korea; khiemduynguyen2000@yahoo.com; 2 Sustainable Management of Natural Resources and Environment Research Group, Faculty of Environment and Labour Safety, Ton Duc Thang University, Ho Chi Minh City, Vietnam; bachquangvu@tdtu.edu.vn; 3 Department of Civil Engineering, Daegu University, 201, Daegudae-ro, Gyeongsan, Gyeongbuk 38453, Korea; jonghan@daegu.ac.kr

**Keywords:** irreversible thermochromic sensors, manganese violet, thermochromic materials, irreversible thermochromic paint, MnNH_4_P_2_O_7_

## Abstract

An irreversible thermochromic material based on manganese violet (MnNH_4_P_2_O_7_) is synthesized. The crystal phase, chemical composition, and morphology of the synthesized material are analyzed using X-ray diffraction, scanning electron microscopy coupled with energy-dispersive X-ray spectrometry, and Fourier-transform infrared spectroscopy. The absorption spectra of the synthesized material are obtained using a UV-Vis spectrometer, and the thermochromism exhibited by the powdered samples at high temperatures is also investigated. The as-synthesized manganese violet pigment consists of pure α-MnNH_4_P_2_O_7_ phase. In addition, the synthesized pigment largely consists of hexagonal crystals with a diameter of hundreds of nanometers. On heating, the pigment simultaneously loses H_2_O and NH_3_ in two successive steps at approximately 330–434.4 °C and 434.4–527 °C, which correspond to the formation of an intermediate phase and of Mn_2_P_4_O_12_, respectively. An overall mass loss of 14.22% is observed, which is consistent with the expected 13.79%. An irreversible color change from violet to white is observed after exposure of the synthesized manganese violet pigment at 400 °C for 30 min. This is attributed to the oxidation of ammonia to hydroxylamine, which then decomposes to nitrogen and water, or alternatively to the direct oxidation of ammonia to nitrogen. Furthermore, we demonstrate the potential application of synthesized manganese violet in the production of irreversible thermochromic paint by mixing with potassium silicate solution as a binder and deionized water as a solvent at a specific ratio. The thermochromic paint is then applied in fabrication of irreversible thermochromic sensors by coating it onto a steel plate surface. Finally, we show that manganese violet-based irreversible thermochromic sensors are able to detect temperatures around 400 °C by changing color from violet to white/milky.

## 1. Introduction

Thermochromic materials are currently receiving considerable attention due to their applications in life sciences [1,2], in flow sensing [3,4,5], as well as their potential applications as temperature sensors in a wide range of devices, such as aeronautical [6,7,8] and gas turbine engine components [9], household appliances [10], hotplates and furnaces [11]. Thermochromic materials can be divided into reversible and irreversible types. The first exhibit a reversible color change following a temperature variation. The change in color can be reversed through heating–cooling cycles, wherein the material regains its original color after cooling [12]. In contrast, irreversible thermochromic materials exhibit an irreversible color change based on the peak temperature of the surrounding environment. The change in color cannot be reversed upon cooling, thus providing permanent records that can be visualized offline [7]. Irreversible color changes over a wide range of temperatures can be obtained by the decomposition of irreversible thermochromic materials such as phosphates, carbonates, and hydroxides [11]. The final color of the irreversible thermochromic material is dependent on both the temperature it is subjected to and the time period over which it is held at a high temperature [9]. Table 1 shows a list of various irreversible thermochromic materials which can change color under different temperatures.

There are many techniques which can be used for surface temperature measurement. Thermocouples are fixed on components during temperature measurements [7]. Their main advantage is to provide very accurate measurements. Hence, they can be used as a valuable means of local validation of the surface temperature. Optic sensors based on fiber Bragg grating technology are another technique for surface temperature measurement. The main advantage of this technique is to give very accurate temperature measurements. However, these measurement techniques only give temperature information at discrete points, and critical hot spots may be missed [7]. Irreversible thermochromic paints are global measurement techniques which give the surface temperature distribution with a high spatial resolution. They can be applied to the most complex surface shapes, and do not interfere with the thermal behavior of the component, as the layer thickness does not exceed 40 μm [7].

Manganese violet (MV, with empirical formula NH_4_MnP_2_O_7_) has been approved for use in a number of important pigment applications, including cosmetics, toys, and plastics [14]. Moreover, the color of this compound changes from violet to white upon heating [13]. In particular, on gentle heating, the color of MV powder reversibly changes from violet to blue because of the loss of water. The original color is restored after cooling in air. Heating to higher temperatures liberates ammonia leaving a white residue, and this color change is irreversible [15].

The first synthesis of MV was reported by Lee and Browne in 1968 [15]; later, the compound was synthesized by Begum and Wright in 2012 to study its detailed structural and physical properties [14]. However, no previous studies have investigated the application of MV in irreversible thermochromic sensors. Additionally, no previous studies have used MV in its reversible range to sense temperature. Therefore, in this study MV powder was prepared to analyze its structural, physical, and thermal properties. The X-ray diffraction (XRD) result revealed the occurrence of pure α-NH_4_MnP_2_O_7_, which was consistent with that of the previous study [14]. The UV-Vis spectrum showed a strong, broad peak at 545 nm, which was due to the ^5^E_g_ → ^5^T_2g_ transition. The thermogravimetry-differential thermal analysis (TGA-DTA) result indicated that an apparent mass loss of both H_2_O and NH_3_ occurred in two successive steps at ~330–434 °C and ~434–527 °C, which agreed well with that of the previous study [14]. Moreover, the synthesized MV powder showed the ability to irreversibly change its color from violet to white upon exposure at around 400 °C for 30 min. The obtained MV pigment was first used to produce irreversible thermochromic paint by mixing with potassium silicate solution as a binder and deionized water as a solvent at a specific ratio. The thermochromic paint was then applied in fabrication of irreversible thermochromic sensors by coating it onto a steel plate surface. These irreversible thermochromic sensors also showed an irreversible color modification from violet to white after exposure at 400 °C for 30 min. This finding highlights the potential applications of MV-based irreversible thermochromic sensors not only for detecting the temperature of engine components, household appliances, furnaces, or high-temperature ovens, but also for the rapid evaluation of the peak temperature of steel bridges or infrastructures after exposure to fire.

## 2. Materials and Methods

### 2.1. Synthesis 

**Figure 1:**
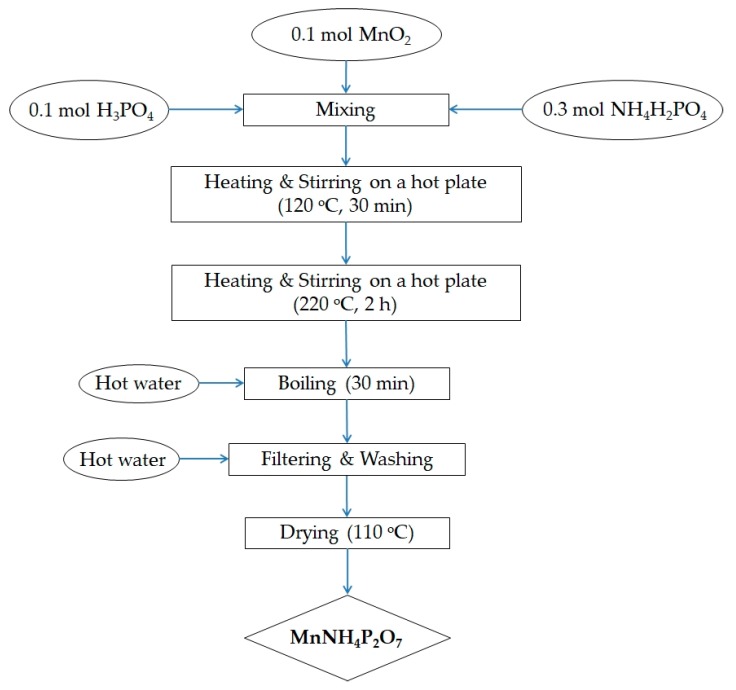
Flow chart for the preparation of manganese violet.

Manganese dioxide (MnO_2_, Sigma-Aldrich, St. Louis, MO, USA) was mixed with ammonium dihydrogen phosphate (NH_4_H_2_PO_4_, Sigma-Aldrich, Tokyo, Japan) and phosphoric acid (H_3_PO_4_, 85.5 wt% in H_2_O, Sigma-Aldrich, Buchs, Switzerland). As the phase of MnO_2_ does not affect the final product, we can use MnO_2_ with any phase for synthesis of MV. The above mixture was heated and stirred on a hot plate at 120 °C for 30 min, and then at 220 °C for a further 2 h. Excess hot water was added to the hot material, and the aqueous suspension was boiled for another 30 min. The product was washed with hot water several times and recovered via vacuum filtration. The synthesized MV powder was dried at 110 °C overnight, and then hand-ground using a pestle in an agate mortar for 30 min. This process yielded a deep violet microcrystalline powder. The synthesis procedure is summarized in Figure 1. Various synthesis conditions were explored, including different reagent ratios, reaction temperatures, and reaction times. It was found that the MV compound could be synthesized by heating and stirring the above reagents, in a Mn–NH_4_–P molar ratio of 1:3:4, up to 220 °C for 2 h.

### 2.2. Characterization Techniques

Power X-ray diffraction (XRD) analysis of the synthesized powder samples was performed on a Rigaku MiniFlex600 diffractometer (Osaka, Japan) with Cu Kα radiation. The powder patterns were recorded at room temperature in a 2θ range from 20 to 50°, with an angular step of 0.02°, and a scanning rate of 2°/min. An external SI standard was used to calibrate the instrument.

The microstructure of the synthesized samples was inspected using scanning electron microscopy (SEM, SUPRA^TM^ 25, Zeiss, Jena, Germany) coupled with energy-dispersive X-ray spectrometry (EDX, X-MAX^N^ (80 mm), Oxford, UK). The powdered samples for microstructural analysis were placed on an aluminum holder and subsequently coated with platinum.

The Fourier-transform infrared (FT-IR) spectra of the samples were characterized by a Nicolet IS5 spectrometer (Thermo Fisher Scientific, Waltham, MA, USA). The specimens were prepared by mixing the powdered samples with dried KBr powder, followed by the application of a force of approximately 25 MPa to form pellets. The spectra were collected over a spectral range of 3600–400 cm^−1^.

The thermal properties of the synthesized powder samples were analyzed by thermogravimetry-differential thermal analysis (TG-DTA, Setaram Setsys Evolution TMA, Caluire, France). The measurements were carried out with milligram-size (10–15 mg) samples in a nitrogen environment, from room temperature to 800 °C, and with a heating rate of 5 °C/min. Variable temperature XRD measurements were also carried out on a Rigaku MiniFlex600 diffractometer with Cu Kα radiation. The samples were prepared by heating to various temperatures from 380 to 420 °C at a rate of 20 °C/min, and maintained at each temperature for 30 min.

The absorption spectra of the samples were obtained using a Konica-Minolta CM-3600d spectrophotometer (Konica Minolta, Osaka, Japan) in a wavelength range from 360 to 740 nm.

The thermochromic color changes of synthesized MV at high temperature were investigated by adding the powdered samples into 5-mL combustion boats, followed by heating to different temperatures from 380 to 420 °C in 10 °C steps in a digital muffle furnace (FX-05, DAIHAN Scientific, Wonju, Korea), and maintaining each temperature for 0, 5, 15, 30, and 60 min. After keeping the powdered samples at different temperatures for different periods, the combustion boats were removed and allowed to cool to room temperature, and the color change of the powder samples was recorded using a digital camera (Canon EOS 100D, Tokyo, Japan)

### 2.3. Thermochromic Sensor Fabrication

**Figure 2:**
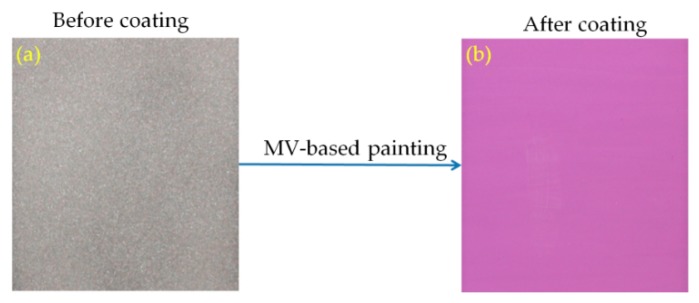
Steel plate specimens before (**a**) and after (**b**) coating.

Irreversible thermochromic sensors were fabricated by coating a thermochromic paint onto the surface of a steel plate substrate (60 × 60 × 2 mm^3^). The paint was produced by mixing the synthesized MV pigment with potassium silicate solution (K_2_SiO_3_, Kanto Chemical, Tokyo, Japan) as a binder and deionized (DI) water as a solvent. The paint was then coated onto the surface of the steel plate specimens using a brush. To improve the cohesion between the coating and the steel plate surface, the latter was roughened by abrasive powder treatment using a sandblasting machine [10]. After air-drying, the coated specimens were stoved in an oven at 300 °C for 1 h to fabricate the thermochromic sensors. Stoving is required to bond the paint film to the substrate; otherwise, the film would have poor toughness and could be easily damaged by handling [16]. Photographs of the steel plate specimens before and after coating are shown in Figure 2a,b. The average thickness of the coating is about 100 µm.

## 3. Results and Discussion

### 3.1. Structure and Properties of Manganese Violet

#### 3.1.1. X-ray Diffraction Spectra

**Figure 3:**
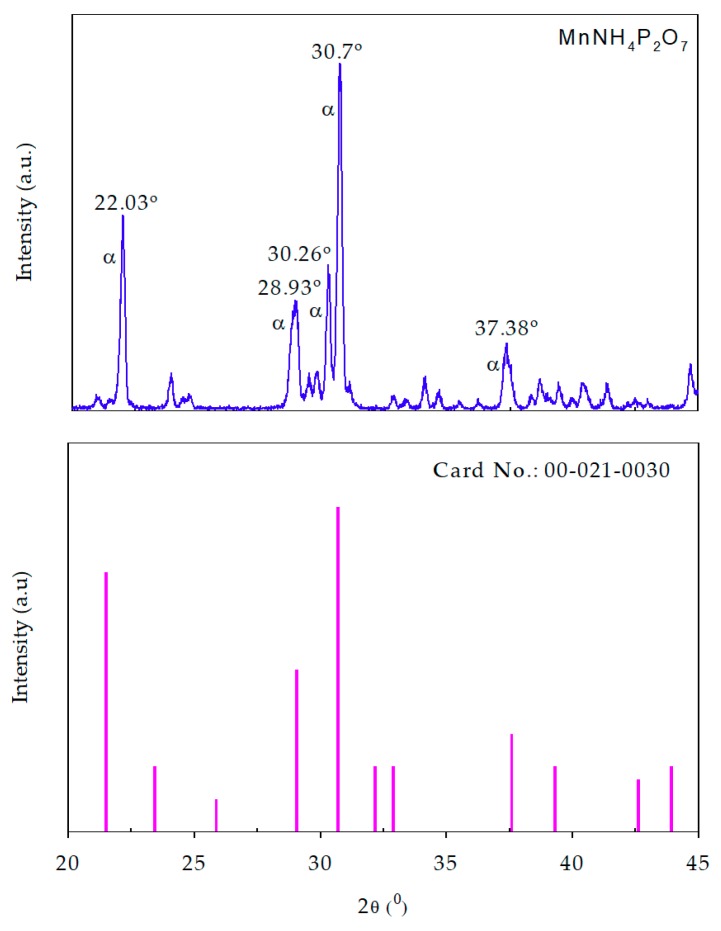
X-Ray Diffraction (XRD) patterns of synthesized manganese violet (**above**) and standard PDF2 pattern (**below**).

Figure 3 shows the XRD patterns of our synthesized MV compound (above) and standard PDF2 pattern (below). Our XRD result agrees well with the pattern of PDF2 Card No.: 00-021-0030. The result also reveals the occurrence of the pure α-MnNH_4_P_2_O_7_ phase in the sample. Recently, Anselmi et al. used the PDF2 database in combination with Rietveld refinement analysis to identify the phase composition of a commercial MV sample obtained from Kremer (45350, Aichstetten, Germany) [17]. However, it was not possible to carry out phase identification of manganese violet by means of the PDF2 database. The XRD analysis revealed that α-MnNH_4_P_2_O_7_ was the most abundant phase in the sample, even though very small amounts of the β-MnNH_4_P_2_O_7_ and kaolinite (Al_2_Si_2_O_5_(OH)_4_) phases were also observed. Moreover, the weight composition of the examined compound was estimated by Rietveld analysis as 79.9% α-MnNH_4_P_2_O_7_, 4.5% β-MnNH_4_P_2_O_7_, 13.3% kaolinite, and 2.3% stewartite (MnFe_2_(PO_4_)_2_(OH)_2_·8H_2_O) [17]. In another study, Begum et al. determined the crystal structure of synthesized MV from Rietveld refinement of neutron powder diffraction data [14]. The refinement analysis confirmed the occurrence of the pure α-MnNH_4_P_2_O_7_ phase in the samples. α-MnNH_4_P_2_O_7_ has a type I pyrophosphate structure, which can be described as a three-dimensional network of MnO_6_ octahedra corner-linked to five P_2_O_7_ units [14]. The XRD result in our study agrees well with that of the previous one [14]. Moreover, it is different from the phase composition of a commercial MV sample obtained from Kremer (45350, Aichstetten, Germany), which contains α-MnNH_4_P_2_O_7_ as the most abundant phase, and very small amounts of the β-MnNH_4_P_2_O_7_ and kaolinite phases; the XRD pattern of our synthesized MV compound reveals the occurrence of pure α-MnNH_4_P_2_O_7_ phase in the sample.

#### 3.1.2. SEM and Spot-Chemical Analysis

**Figure 4:**
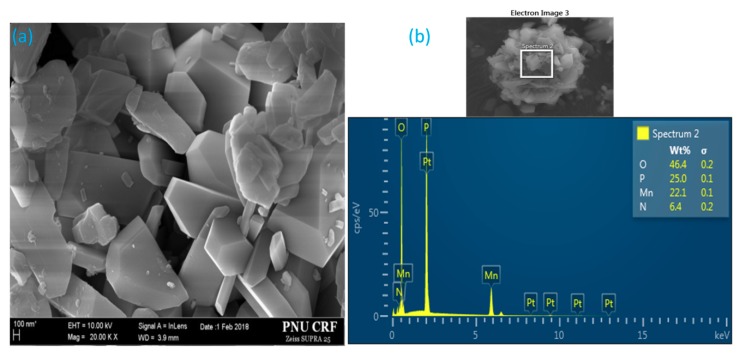
(**a**) SEM micrograph and (**b**) EDX analysis of synthesized manganese violet.

Figure 4a and Figure S1 (Supplementary Materials) illustrate the microstructure of the as-prepared MV powder. The SEM micrographs clearly show that the synthesized MV pigment largely consisted of hexagonal crystals with a diameter of hundreds of nanometers. The random spot-chemical EDX analysis confirmed the presence of four main elements in the system (Figure 4b). The inset table in the figure shows the elemental content of the synthesized sample. The mass contents of O, P, Mn, and N were 46.4%, 25.0%, 22.1%, and 6.4%, respectively, while the corresponding mass contents of Mn^3+^, NH_4_^+^, and P_2_O_7_^4−^ were 22.1%, 8.23%, and 70.16%, respectively. However, the above contents of O, P, Mn, and N, are probably not accurate due to the errors in oxygen and nitrogen determination.

#### 3.2.3. FT-IR Spectra

**Figure 5:**
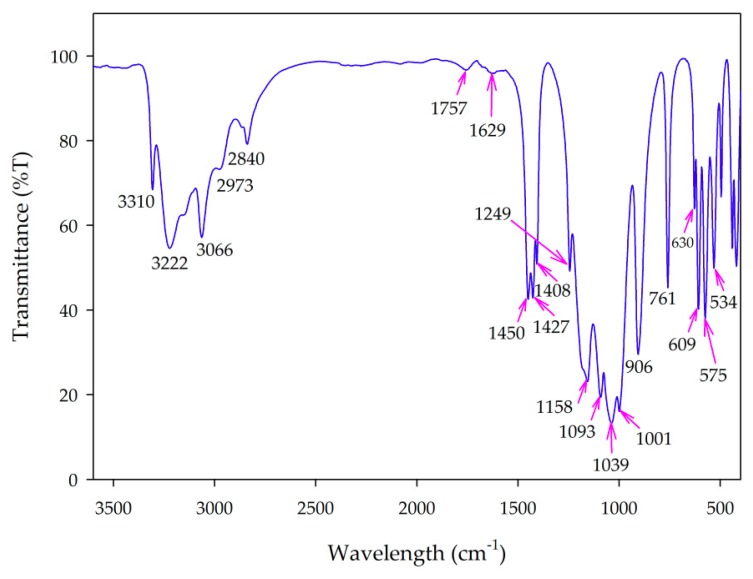
Fourier-transform infrared (FT-IR) spectrum of synthesized manganese violet.

Figure 5 shows the FT-IR spectrum of the synthesized MV powder, which highlights several interesting features. Broad bands in the 3310–2840 cm^−1^ region correspond to the stretching vibrations of both H_2_O molecules and NH_4_^+^ ions [18,19,20]; the band broadening can be attributed to hydrogen bonding within the crystal [21]. The bands in the 1757–1629 cm^−1^ region correspond to the bending vibrations of H_2_O and NH_4_^+^ ions. The bending vibration of NH_4_^+^ is also observed in the 1450–1408 cm^−1^ range [18,22]. The asymmetric and symmetric terminal stretching vibrations of PO_3_ groups usually occur in the 1249–1001 cm^−1^ region [23]. The two peaks at 906 cm^−1^ and 761 cm^−1^ are attributed to the vibrations of P–O–P bridges [18], whereas the bands in the 630–534 cm^−1^ region are due to the vibrations of the diphosphate groups. This result, together with XRD and SEM-EDX results, demonstrated the formation of α-MnNH_4_P_2_O_7_ pigment.

### 3.2. Thermal Properties

**Figure 6:**
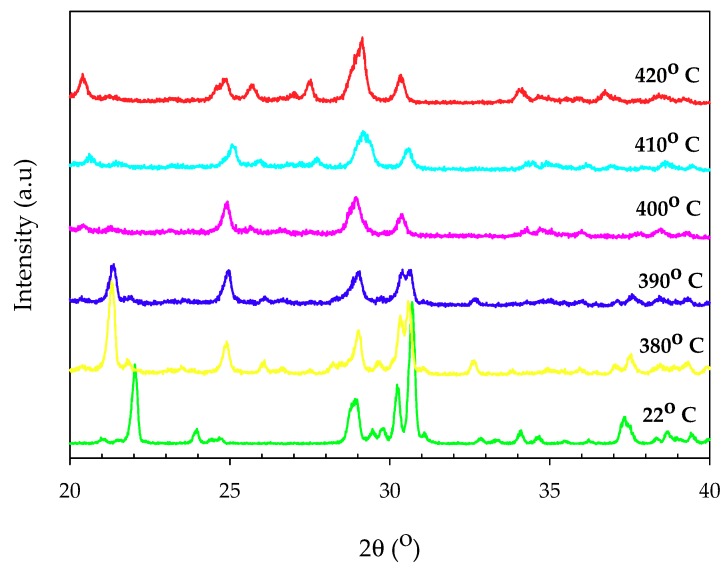
Variable temperature XRD of α-MnNH_4_P_2_O_7_.

The XRD pattern of thermal annealing samples (Figure 6) showed that α-MnNH_4_P_2_O_7_ began to lose crystallinity at 380 °C, with extra peaks evident in the diffraction pattern. From 400 °C, crystalline α-MnNH_4_P_2_O_7_ was lost, leaving an unidentified poorly crystalline material up to 420 °C. The disappearance of peaks around 22° and 37.4°, together with the suppression of peak around 30.7°, demonstrated the thermal decomposition of α-MnNH_4_P_2_O_7_. On the other hand, the appearance of a peak around 24.9° indicated the formation of an intermediate phase, and of Mn_2_P_4_O_12_ [14].

**Figure 7:**
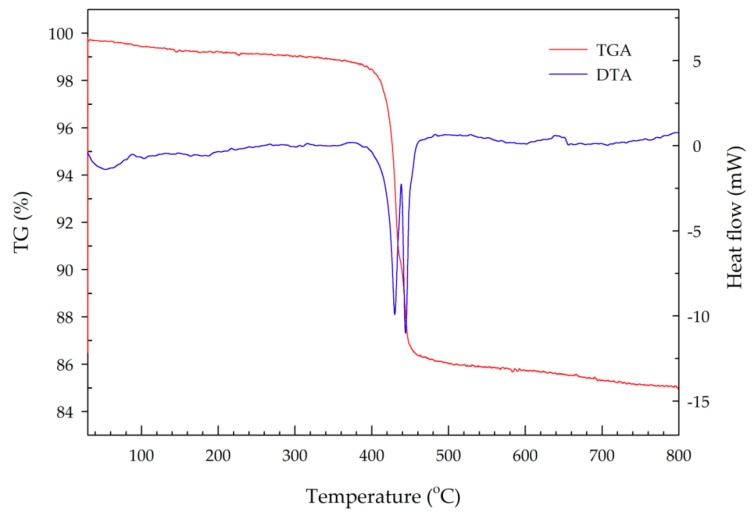
TGA-DTA plot of synthesized manganese violet.

The TGA-DTA plot given in Figure 7 indicated that an apparent mass loss of both H_2_O and NH_3_ occurred in two successive steps at ~330–434.4 °C and 434.4–527 °C, which corresponds to the formation of an intermediate phase and of Mn_2_P_4_O_12_, respectively [14,15]. An overall mass loss of 14.22% was observed, which is consistent with the expected 13.79%. Lee and co-workers analyzed the thermal decomposition of synthesized MV using a Stanton recording thermobalance [15]. They found that the decomposition of MnNH_4_P_2_O_7_ proceeded in two stages. The first stage occurred between 150 and 340 °C, and corresponded to the formation of the Mn_2_P_4_O_13_(NH_3_)_2_ compound (blue color). A mass loss of about 3% of the total weight was observed, reflecting the evaporation of water. This compound and its blue color are unstable. On cooling in air, it absorbs water from the air and restores the original violet color. The second stage occurred between 340 and 460 °C, corresponding to the formation of a manganous tetrametaphosphate compound (Mn_2_P_4_O_12_, white color). In this stage, a very sharp mass loss of 10% of the total weight was observed, due to the oxidation of ammonia to hydroxylamine, which then decomposed to nitrogen and water [15]. In another study, Begum and coworkers studied the thermal stability of synthesized MnNH_4_P_2_O_7_ using thermogravimetric analysis/mass spectrometry (TGA-MS). They found that simultaneous mass loss of H_2_O and NH_3_ occurred in two successive steps, at approximately 340–436 °C and then 436–520 °C, which correspond to the formation of an intermediate phase and of Mn_2_P_4_O_12_, respectively [14].

### 3.3. UV-Vis Spectra

**Figure 8:**
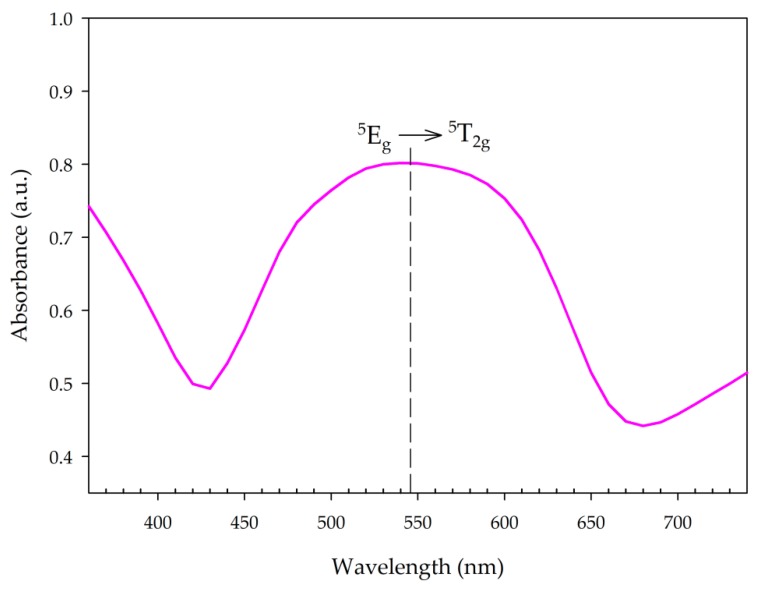
UV-Vis spectrum of synthesized manganese violet.

The structure of MnNH_4_P_2_O_7_ can be described as a three-dimensional network of MnO_6_ octahedra corner-linked to five P_2_O_7_ units, in which Mn^3+^ exists as a high-spin d^4^ ion; in a regular octahedral coordination environment, this ion would be expected to exhibit an absorbance band in the visible region due to the ^5^E_g_ → ^5^T_2g_ transition. Figure 8 shows the absorbance spectrum of synthesized MV samples. The electronic spectrum shows a strong, broad peak at 545 nm, which is consistent with previous data [14,24].

### 3.4. Thermochromism of Synthesized Pigment Powders

**Figure 9:**
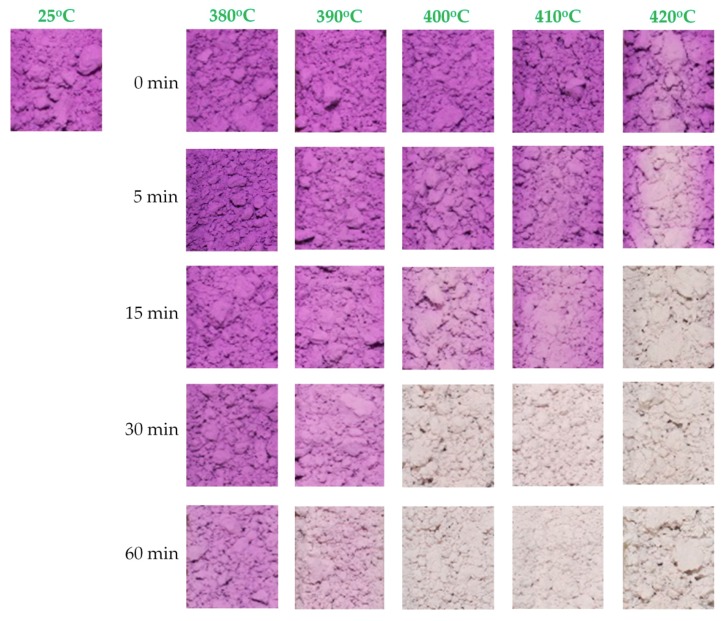
Color of synthesized manganese violet samples exposed at different temperatures for different periods after cooling.

After synthesizing the MV pigment and analyzing its structural properties, the thermochromism of the synthesized powders was also studied. As discussed in the Experimental section, the thermochromic color change of the synthesized samples at high temperature was investigated by placing the powdered samples into 5-mL combustion boats. Subsequently, the samples were heated to different temperatures in a digital muffle furnace at a rate of 20 °C/min, and maintained at each temperature for 0, 5, 15, 30, and 60 min. After exposing the powdered samples to different temperatures for different periods, the combustion boats were removed and allowed to cool to room temperature for 1 h, while recording the color at specific time points. The results are shown in Figure 9. At 380 °C, the color change of the powdered samples was not clear and insufficient to be observed by the naked eye, regardless of the exposure time. At 390 °C, the color of MV changed from violet to light pink after exposure for 60 min. At 400 °C, the color of MV started changing from violet to light pink after exposure for 15 min, and completely changed to white after exposure for 30 min. At 420 °C, the color of MV completely changed from violet to white after only 15 min of exposure time. This is due to the higher speed of oxidation and decomposition at higher temperature. No further color changes were observed, even after increasing the exposure time to 60 min. After cooling to room temperature, the powdered samples remained white in color, and did not recover their original color. This demonstrated that the color of MV changes from violet to white upon exposure at around 400 °C for 30 min, and this color change is irreversible. The color change at high temperature can be attributed to the oxidation of ammonia to hydroxylamine, which then decomposes to nitrogen and water, or alternatively to the direct oxidation of ammonia to nitrogen [10]. After oxidation followed by decomposition, the chemical formula of MV changes from MnNH_4_P_2_O_7_ to Mn_2_P_4_O_12_. The resulting material is not able to recover its original structure after cooling. This effect can explain the irreversible color change properties of the MV compound.

### 3.5. Manganese Violet-Based Irreversible Thermochromic Sensors

**Figure 10:**
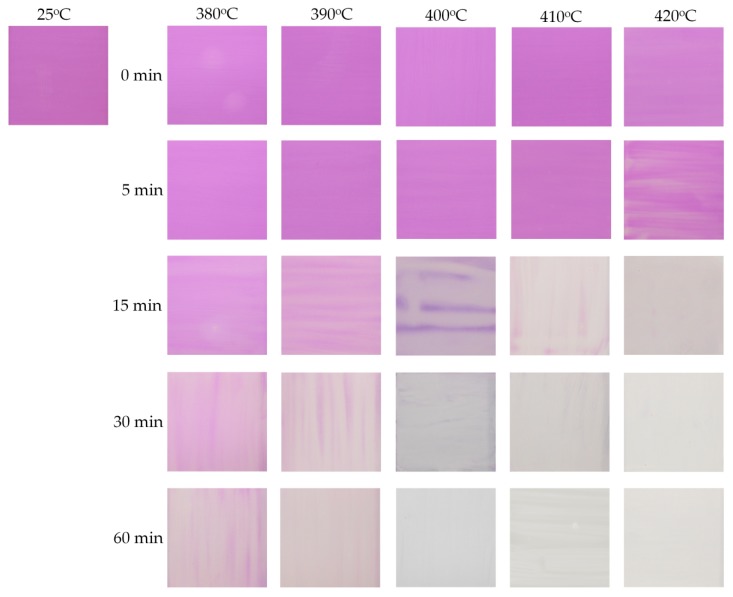
Color changes of sensor samples exposed at different temperatures for different periods after cooling.

Irreversible thermochromic sensors were fabricated by coating a MV-based thermochromic paint onto a steel plate substrate. After drying, the adhesion and resistance to abrasion of the paint film were tested by scratching on the dried surface with a finger. Various mixing conditions were explored, including different reagent ratios and stirring times. It was found that the best thermochromic paint, with excellent adhesion and resistance to abrasion, could be obtained by mixing and stirring the reagents in a MV pigment–potassium silicate–DI water weight ratio of 40:5:55, at room temperature for 30 min. Briefly, 40 g of synthesized MV was mixed with 5 g of potassium silicate solution and 55 g of DI water. The mixture was vigorously stirred for 30 min at room temperature. The resulting paint was then deposited onto the treated steel plate surface with a brush, and then dried. A second layer of the resulting paint was then deposited onto the first dried layer, and then dried. In order to elucidate the relationship between color and temperature, the sensor plates were individually heated to different temperatures in a digital muffle furnace, and maintained at each temperature for 0, 5, 15, 30, and 60 min. The process was repeated for a number of samples at different temperatures, to cover the whole measurement range from 380 to 420 °C in 10 °C steps. The color change of the sensor plates was recorded after cooling to room temperature using a digital camera (Canon EOS 100D). Figure 10 shows the color changes observed for the sensor samples at different temperatures for different periods. The original color of the sensor was violet, and started to change to light pink after exposure at 380 or 390 °C for 60 min. At 400 °C, the color of the sensor plate completely changed to white/milky after exposure for 30 min. At higher temperature (e.g., 420 °C), the color change of MV occurred faster due to the higher speed of oxidation and decomposition. No further color changes were observed, even after increasing the exposure time to 60 min. After cooling to room temperature, the sensor samples remained white and did not recover the original violet color. This demonstrated that the color of the sensor changes from violet to white/milky upon exposure at around 400 °C for 30 min, and this color change is irreversible. In thermochromic paints, the pigments are responsible for the thermochromic properties of the coating, while the binder facilitates the cohesion between the pigment particles, and the solvent helps in dissolving the binders and dispersing the pigments [7]. Thus, the color change of the present thermochromic sensor at high temperature can be attributed to the decomposition of the MV pigment, resulting in the formation of a new compound in the paint. At high temperature, the ammonia component of the MV pigment (MnNH_4_P_2_O_7_) is oxidized to hydroxylamine, which then decomposes to nitrogen and water, resulting in the formation of Mn_2_P_4_O_12_ [15]. This causes the irreversible color change of the MV pigment. This finding demonstrates that the visible color change of MV from violet to white/milky is irreversible and occurs around 400 °C, suggesting that the synthesized MV material could be applied in irreversible thermochromic sensors to monitor temperature changes around 400 °C.

## 4. Conclusions

In summary, we have successfully synthesized and characterized the structure and properties of the MV pigment. The as-synthesized pigment consists of pure α-MnNH_4_P_2_O_7_ phase and rod-shaped crystals with a diameter of hundreds of nanometers. On heating, the pigment simultaneously loses H_2_O and NH_3_ in two successive steps at approximately 330–434.4 °C and 434.4–527 °C. An irreversible color change of the synthesized MV pigment from violet to white/milky is observed after exposure to 400 °C for 30 min. In addition, the observed thermal behavior of a MV-based thermochromic sensor highlights the potential of this system for temperature sensing applications. This study shows that the synthesized MV pigments are promising smart materials for applications in irreversible thermochromic sensors, not only to detect the temperature of engine components, household appliances, furnaces, or high temperature ovens, but also for the rapid evaluation of the peak temperature of steel bridges or infrastructures after exposure to fire.

## Figures and Tables

**Table 1:** Thermochromic materials, their reversible (↔) or irreversible (→) color change, and transition temperatures [13].

Thermochromic Materials	Color Change	Transition Temperature (°C)
[NH_2_(C_2_H_5_)_2_]_2_CuCl_4_	deep green ↔ yellow	38
Ag_2_(HgI_4_)	yellow ↔ orange	50
CuI	gray-tan → orange	60–62
Cu_2_(HgI_4_)	red ↔ brown	70
HgI_2_	red ↔ yellow	127
2Cu(CNS)_2_·2pyridine	green → yellow	135
	yellow → black	220
NH_4_VO_3_	white → brown	150
	brown → black	170
CoCO_2_	violet → black	330
MnNH_4_P_2_O_7_	violet → white	400
NiC_2_O_4_	light blue → black	410

## Supplementary Materials

The following are available online at http://www.mdpi.com/1996-1944/11/9/1693/s1. Figure S1: SEM micrographs of synthesized manganese violet pigment at different magnifications: 5 k×, 10 k×, and 20 k×.
