# Supplementary material for: Synthesis and Irreversible Thermochromic Sensor Applications of Manganese Violet
Source: Materials (Basel). 2018 Sep 12;11(9):1693. doi: 10.3390/ma11091693 (PMC6165037; doi:10.3390/ma11091693)
Supplement: Supplementary file 1 [file materials-11-01693-s001.pdf]

Supplementary Materials

Synthesis and Irreversible Thermochromic Sensor Applications of Manganese Violet

Duy Khiem Nguyen [1], Quang-Vu Bach [2], Jong-Han Lee [3], and In-Tae Kim [1,*]

[1] Department of Civil Engineering, Pusan National University, 2, Busandaehak-ro 63beon-gil, Geumjeong-gu, Busan, 46241, Republic of Korea; khiemduynguyen2000@yahoo.com
[2] Sustainable Management of Natural Resources and Environment Research group, Faculty of Environment and Labour Safety, Ton Duc Thang University, Ho Chi Minh City, Vietnam; bachquangvu@tdtu.edu.vn
[3] Department of Civil Engineering, Daegu University, 201, Daegudae-ro, Gyeongsan, Gyeongbuk 38453, Republic of Korea; jonghan@daegu.ac.kr
* Correspondence: itkim@pusan.ac.kr; Tel.: +82-51-510-2497; Fax: +82-51-513-9596

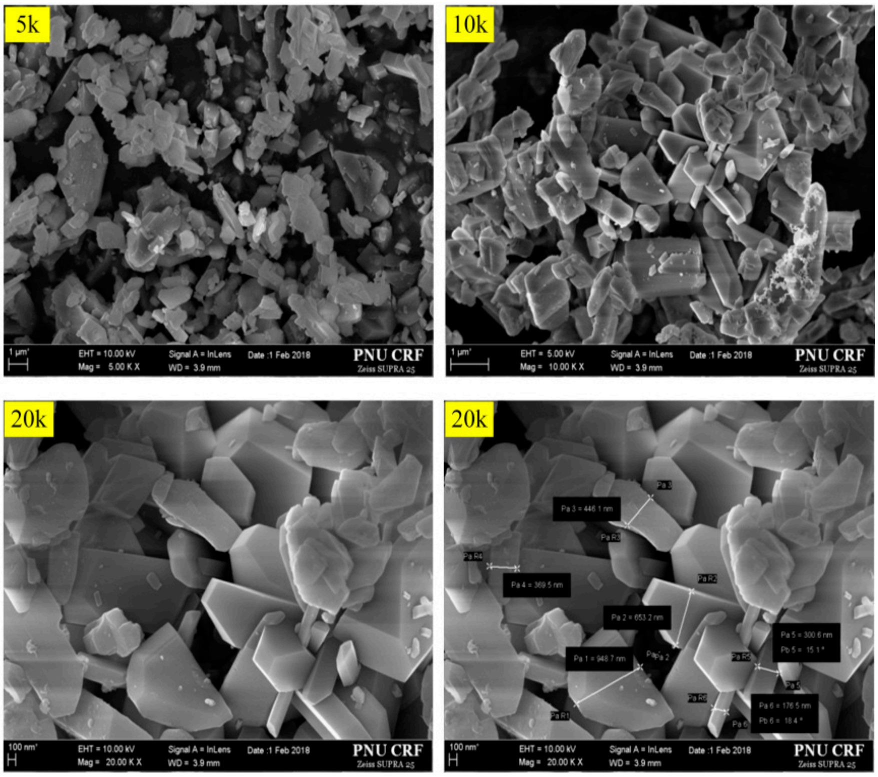

Figure S1. SEM micrographs of synthesized manganese violet pigment at different magnifications: 5 k×, 10 k×, and 20 k×.
